# Challenges in undertaking nonlinear Mendelian randomization

**DOI:** 10.1002/oby.23927

**Published:** 2023-10-16

**Authors:** Kaitlin H. Wade, Fergus W. Hamilton, David Carslake, Naveed Sattar, George Davey Smith, Nicholas J. Timpson

**Affiliations:** ^1^ Medical Research Council (MRC) Integrative Epidemiology Unit University of Bristol Bristol UK; ^2^ Population Health Sciences, Bristol Medical School, Faculty of Health Sciences University of Bristol Bristol UK; ^3^ Infection Science North Bristol NHS Trust Bristol UK; ^4^ Institute of Cardiovascular and Medical Sciences, British Heart Foundation Glasgow Cardiovascular Research Centre University of Glasgow Glasgow UK

## Abstract

Mendelian randomization (MR) is a widely used method that exploits the unique properties of germline genetic variation to strengthen causal inference in relationships between exposures and outcomes. Nonlinear MR allows estimation of the shape of these relationships. In a previous paper, the authors applied linear and nonlinear MR to estimate the effect of BMI on mortality in UK Biobank, providing evidence for a J‐shaped association. However, it is now clear that there are problems with widely used nonlinear MR methods, which draws attention to the likely erroneous nature of the conclusions regarding the shapes of several explored relationships. Here, the authors explore the utility and likely biases of these nonlinear MR methods with the use of a negative control design. Although there remains good evidence for a causal effect of higher BMI increasing the risk of mortality, the pattern of this association across different levels of BMI requires further characterization.

Mendelian randomization (MR) is a widely used method that exploits the unique properties of germline genetic variation to strengthen causal inference in observational epidemiological studies [1]. There have been many developments in MR methodology that have expanded the scope of what can be estimated and have provided many sensitivity analyses that facilitate robust inference. One of these developments is nonlinear MR, which apparently allows estimation of the shape of the relationship between an exposure and outcome [2]. One observational epidemiological association that is often reported as nonlinear is that between body mass index (BMI) and mortality, which is markedly J‐shaped. In a paper published in *Obesity*, we applied linear and nonlinear MR to estimate the effect of BMI on mortality in UK Biobank [3]. Using a linear MR model, there was evidence for a causal effect of BMI on the risk of both all‐cause mortality and mortality from specific causes, including cardiovascular disease (CVD). For example, the hazard increased by approximately 3% (hazard ratio: 1.03; 95% confidence interval [CI]: 0.99–1.07) for all‐cause mortality and by approximately 10% (hazard ratio: 1.10; 95% CI: 1.01–1.19) for mortality from CVD with every kilogram per meter squared higher BMI.

In the same work, the then relatively new nonlinear MR approach [2] was applied to characterize the shape of the associations. This method involves calculating the residuals from regression of measured BMI on the genetic instrument and undertaking MR analyses in strata defined by the “instrument‐free” residualized BMI. This avoids the collider bias that would be generated by simply stratifying on measured BMI [2]. In these analyses, there was weak evidence for a J‐shaped association between BMI and mortality. Indeed, a later analysis using both UK Biobank and the Nord‐Trondelag Health (HUNT) study found similar results using the same method, although these results were likely complicated by additional stratification by smoking status [4], which itself could cause collider bias because BMI influences smoking behavior [5].

It is now evident that there are serious problems with this widely used nonlinear MR method [6, 7]. In an application to vitamin D and mortality [8], impossible findings were generated [7]. The authors of the residualization method fully accept that the strikingly nonlinear results generated are spurious [6], and the journal has issued an “expression of concern” regarding the paper [9].

It appears highly likely that errors will be generated in other applications of the nonlinear MR approach, which has led us to draw attention to the likely erroneous nature of the conclusions regarding the shape of the relationship between BMI and mortality in our paper. We note, however, that our conclusions regarding the conventional MR finding remain unchanged. To uncover potential problems with the nonlinear MR approach, we have applied a negative control methodology, which uses a factor in the analyses as the outcome that cannot possibly be altered by the exposure in question [10]. It is clear that BMI cannot influence assigned sex; therefore, we applied the same nonlinear MR analytical strategy that we had used in our paper in a comparable sample of UK Biobank, but replaced mortality with assigned sex as the outcome (Figure 1). The figure shows that the method produces the nonsensical finding that lower BMI makes participants less likely to be male, whereas high BMI renders them more likely to be male. Such bias may be induced by issues such as nonconstant genetic effects across the range of BMI or differential selection bias within the strata of residual BMI, among other possibilities.

**FIGURE 1:**
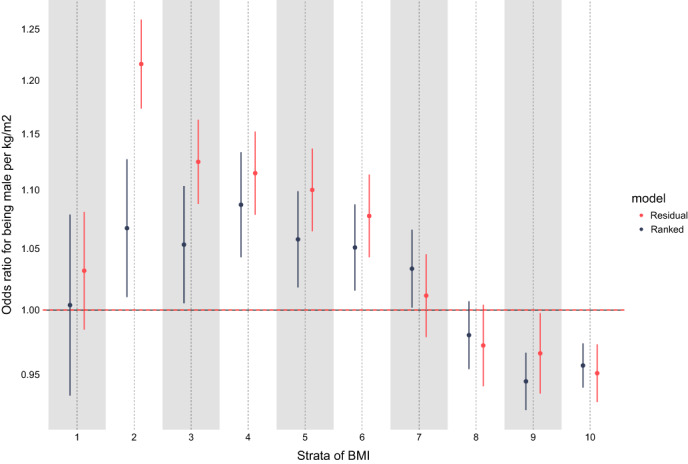
Mendelian randomization estimates from the negative control study showing the “causal effect” of BMI on assigned sex within strata of BMI using UK Biobank data comparing the residual and doubly ranked methods. Points on the figure represent odds ratios for being male with each kilogram per meter squared higher BMI, and error bars represent respective 95% CIs. Here, to avoid possible bias, the analysis was conducted among people in whom their self‐reported sex was consistent with their sex chromosomes (i.e., XY for males and XX for females), where the reference was “female.” After calculating the residual or ranked BMI distribution for the application of each method, BMI strata were defined by splitting the resulting continuous residual or ranked BMI distribution into 10 equal quantiles.

In their letter acknowledging that their residualization method was flawed, the authors advanced a supposedly more robust “doubly ranked” nonlinear MR approach [6]. Our figure shows that this also fails a sanity check using assigned sex as a negative control outcome [10].

When applying conventional MR methodology (i.e., assuming linearity in the relationships between the genetic instrument(s) and the exposure and between the exposure and outcome), the relationship between BMI and assigned sex was far weaker (odds ratio for being male with each kilogram per meter squared higher BMI: 1.02; 95% CI: 1.01–1.03). This is in clear contrast to the magnitude of estimates provided within BMI strata (Table 1) and the coincident heterogeneity in estimates across strata of BMI in the nonlinear MR analyses (where heterogeneity *p* values across strata for both residual and ranked methods were <2e‐16). Nevertheless, these results continue to highlight the requirement for careful interpretation of imperfect MR estimates in light of complexity in polygenic instrumentation, measurement and sampling frames.

**TABLE 1:** LACE estimates using MR from the negative control study showing the “causal effect” of BMI on assigned sex within strata of BMI using UK Biobank data comparing the residual and ranked methods

Strata of BMI	β	SE	LCI	UCI	*p*
**Residual**					
**1**	0.03	0.02	−0.02	0.08	0.19
**2**	0.20	0.02	0.16	0.23	2.49 × 10^−27^
**3**	0.12	0.02	0.08	0.15	5.38 × 10^−12^
**4**	0.11	0.02	0.08	0.14	1.09 × 10^−10^
**5**	0.10	0.02	0.06	0.13	1.27 × 10^−08^
**6**	0.07	0.02	0.04	0.11	8.24 × 10^−06^
**7**	0.01	0.02	−0.02	0.04	0.49
**8**	−0.03	0.02	−0.06	0.004	0.09
**9**	−0.03	0.02	−0.07	−0.002	0.03
**10**	−0.05	0.01	−0.07	−0.03	1.77 × 10^−05^
**Ranked**					
**1**	0.004	0.04	−0.07	0.08	0.91
**2**	0.07	0.03	0.01	0.12	0.02
**3**	0.05	0.02	0.01	0.10	0.03
**4**	0.08	0.02	0.04	0.13	8.36 × 10^−05^
**5**	0.06	0.02	0.02	0.09	0.004
**6**	0.05	0.02	0.02	0.08	0.004
**7**	0.03	0.02	0.002	0.06	0.04
**8**	−0.02	0.01	−0.05	0.01	0.15
**9**	−0.06	0.01	−0.08	−0.03	1.06 × 10^−06^
**10**	−0.04	0.01	−0.06	−0.03	8.51 × 10^−07^

*Note*: β represent log odds ratios for being male with each kilogram per meter squared higher BMI. Here, to avoid possible bias, the analysis was conducted among people where their self‐reported sex was consistent with their sex chromosomes (i.e., XY for males and XX for females), where the reference was “female.” After calculating the residual or ranked BMI distribution for the application of each method, BMI strata were defined by splitting the resulting continuous residual or ranked BMI distribution into 10 equal quantiles.

Abbreviations: LACE, local average causal effect; LCI, lower confidence interval; MR, Mendelian randomization; UCI, upper confidence interval.

Due to ongoing concern about the reliability of the nonlinear MR methodology that we focused on here (flagged by the extent of likely biases generated) and in the absence of a definitive answer as to the magnitude of impact biases of this nature elsewhere, we feel it necessary to state that the nonlinear MR findings included in our original paper, published in *Obesity*, may well be unreliable. Unfortunately, we think the same concern applies to the findings in the >70 other papers that have been published using this method.

The application and interpretation of advanced statistical methods often require many assumptions to be met. Although there remains good evidence for a causal effect of higher BMI increasing the risk of mortality, the pattern of this association across different levels of BMI requires further characterization. Currently, the exact source of error generated by both the original and the newly proposed nonlinear MR methods is unclear, and we suggest there should be a moratorium on further applications of them until there is better evidence regarding reliability. This example provides yet more evidence as to why triangulation of evidence across multiple epidemiological study designs with orthogonal biases is necessary for reliable interpretation. In this case, the use of a negative control outcome casts the findings into doubt, as it would have done for the impossible results that were published regarding vitamin D and mortality.

## CONFLICT OF INTEREST STATEMENT

The authors declared no conflict of interest.

## References

[1] Richmond RC , Davey Smith G . Mendelian randomization: concepts and scope. Cold Spring Harb Perspect Med. 2022;12:a040501. 34426474 10.1101/cshperspect.a040501 PMC8725623

[2] Staley JR , Burgess S . Semiparametric methods for estimation of a nonlinear exposure‐outcome relationship using instrumental variables with application to Mendelian randomization. Genet Epidemiol. 2017;41:341‐352. 28317167 10.1002/gepi.22041 PMC5400068

[3] Wade KH , Carslake D , Sattar N , Davey Smith G , Timpson NJ . BMI and mortality in UK Biobank: revised estimates using Mendelian randomization. Obesity (Silver Spring). 2018;26:1796‐1806. 30358150 10.1002/oby.22313 PMC6334168

[4] Sun Y‐Q , Burgess S , Staley JR , et al. Body mass index and all cause mortality in HUNT and UK Biobank studies: linear and non‐linear Mendelian randomisation analyses. BMJ. 2019;364:l1042. 30957776 10.1136/bmj.l1042 PMC6434515

[5] Carreras‐Torres R , Johansson M , Haycock PC , et al. Role of obesity in smoking behaviour: Mendelian randomisation study in UK Biobank. BMJ. 2018;361:k1767. 29769355 10.1136/bmj.k1767 PMC5953237

[6] Burgess S , Wood AM , Butterworth AS . Mendelian randomisation and vitamin D: the importance of model assumptions–Authors' reply. Lancet Diabetes Endocrinol. 2023;11:15‐16. 36528346 10.1016/S2213-8587(22)00344-8

[7] Davey Smith G . Mendelian randomisation and vitamin D: the importance of model assumptions. Lancet Diabetes Endocrinol. 2023;11:14. 10.1016/S2213-8587(22)00345-X 36528345

[8] Sofianopoulou E , Kaptoge SK , Afzal S , et al. Estimating dose‐respons relationships for vitamin D with coronary heart disease, stroke, and all‐cause mortality: observational and Mendelian randomisation analyses. Lancet Diabetes Endocrinol. 2021;9:837‐846. 34717822 10.1016/S2213-8587(21)00263-1 PMC8600124

[9] Expression of Concern—Estimating dose‐response relationships for vitamin D with coronary heart disease, stroke, and all‐cause mortality: observational and Mendelian randomisation analyses. Lancet Diabetes Endocrinol. 2023;11:634. doi:10.1016/S2213-8587(23)00198-5 37454668 PMC10563129

[10] Hamilton FW , Hughes DA , Spiller W , Tilling K , Davey Smith G . Non‐linear Mendelian randomization: evaluation of biases using negative controls with a focus on BMI and vitamin D. medRxiv 2023. doi:10.1101/2023.08.21.23293658 PMC11219394 38789826

